# Study on energy evolution and crack propagation of filling mortar-rock at different loading rates

**DOI:** 10.1371/journal.pone.0327902

**Published:** 2025-07-29

**Authors:** Hanqiu Wang, Chengyong Liu, Yuyi Wu, Yuhua Guan, Tongde Zhao

**Affiliations:** 1 China Coal Energy Research Institute Co., Ltd, Xi’an, Shanxi, PR China; 2 Shandong Huaxin Construction Engineering Group Co., Ltd., Taian, Shandong, PR China; 3 School of Mechanics and Civil Engineering, China University of Mining and Technology(Beijing), Beijing, PR China; Jazan University College of Engineering, SAUDI ARABIA

## Abstract

Shotcrete, as a highly efficient reinforcement material widely used in geotechnical engineering, demonstrates irreplaceable advantages in projects such as tunnel excavation, mine roadway support, and slope protection. However, when shotcrete becomes tightly bonded with rock masses, the energy evolution and crack initiation mechanisms between the two materials exhibit remarkable complexity. Different loading rates significantly alter the internal stress distribution and deformation characteristics within the composite system, thereby influencing the patterns of energy evolution and crack propagation. Consequently, it is essential to investigate the mechanical behavior of filling mortar-rock under varying loading rates. Firstly, uniaxial tests with four loading rates were conducted for the composite specimens, and the effects of loading rate on the mechanical parameters, energy evolution and fracture modes were analyzed. The results show that the mechanical parameters of the composite decrease with the rise of loading rate, and the decrease reaches the maximum when the mortar strength is M20. All three types of energies decreased exponentially with increasing loading rate. The decrease reaches the maximum at a mortar strength of M40. Subsequently, a damage model applicable to the composite specimens was established based on the development rules of the dissipated energy and the compaction coefficient. Finally, PFC^2D^ was used to simulate and analyze the specimens with mortar grade of M30 to investigate the crack propagation and stress evolution process at four loading rates. The results show that tensile stress is the causative factor of crack propagation. The cracks first appeared at the interface, and were mainly distributed on both sides of the specimen after cracking.

## Introduction

During tunnel construction, shotcrete is often used as a reinforcement material in the design of rock reinforcement to enhance the stability of rock, which is bonded together with rock to form a composite [1–4]. The composite specimens are composed of two materials combined together, and there is an interface, this results in the mechanical properties of the specimens becoming highly intricate. Compared with intact rocks, the fracture mechanism and mechanical response of the composite under loads are much more complicated [5–7]. Not only the material properties and size, but also factors such as the roughness and angle of the contact surface should be considered.

In recent years, impact splitting experiments on filling mortar-rock composites were conducted extensively to gain a more comprehensive understanding of the tensile properties of the composites. Li et al. [8] carried out dynamic fracture tests on mortar-granite to explore the impact of interface parameters on dynamic mechanical parameters. The results show that the change of interface parameters will lead to different fracture mechanisms. Qiu et al. [9–10] used Autodyn and Abaqus to carry out impact splitting tests on mortar-granite specimens, analyzed the crack propagation process at the interface. Wu et al. [11] established a new virtual crack model based on the experimental results of mortar-rock, and explored the relationship between stress and crack width at the interface. Guo et al. [12] made rock-concrete specimens with interface cracks, and used DIC technology to demonstrate the crack propagation process under different temperatures and interface crack inclination angles in detail. The results show that the crack propagation path and fracture energy at different crack angles are significantly affected by temperature.

In fact, not all the interfaces of the assemblies are flat, and the assemblies with serrated angles are also common in practical engineering, and shear tests of assemblies are often carried out. Zhao et al. [13] conducted indoor direct shear tests and numerical analysis on mortar-rock specimens, and discussed in detail the effect of sawtooth angles on interface stiffness and displacement. Tang et al. [14–15] used the acoustic emission system for auxiliary monitoring during the direct shear tests, determined three acoustic emission parameters applicable to structural stability analysis. Wang et al. [16] conducted experimental research on filling mortar-rock specimens at high loading rates. An interface slip model of the composite body suitable for high loading rates was established, and the rates mechanism of interface slip was revealed. Badika et al. [17] revealed the effect of roughness on the shear behavior of rock-concrete interface by using cohesive friction model.

Additionally, the deep rock is often subjected to different types of pressure, and the failure of rock under loads shows different characteristics. Shi et al. [18] made concrete and different types of rocks into composites and carried out triaxial compression tests to examine the relationships between confining pressure and mechanical behaviors of the composite body, and established a model to describe dilatancy angle of the composite body according to the experimental parameters. Chang et al. [19] simulated the crack propagation rules of concrete-rock with double cracks under static loads by coupling finite element and discrete element. The results show that there is a strength ratio of concrete to rock, which is closely related to the crack propagation mechanism. Han et al. [20] performed dynamic tests on rock-mortar with varying degrees of crack stiffness and roughness. The results show that tensile cracks are prone to initiating at the interface.

However, the above studies are keen to analyze the mechanical behavior of the composite under different loads and combination modes, while the static energy evolution rules and crack propagation mechanisms of composites under different loading rates are less explored. As a kind of common composites, filling mortar-sandstone composite material will be loaded at different rates by blasting and tunneling in engineering construction, and the corresponding energy evolution of the composite will be different. Therefore, it is imperative to clarify the energy evolution rules of the composite under different loading rates. In this paper, different types of composites were subjected to static loads at four loading rates to analyze the corresponding energy development rules, and then PFC^2D^ was used to explore the crack propagation mechanism of the composites.

### Specimen preparation and test procedure

**Fig 1:**
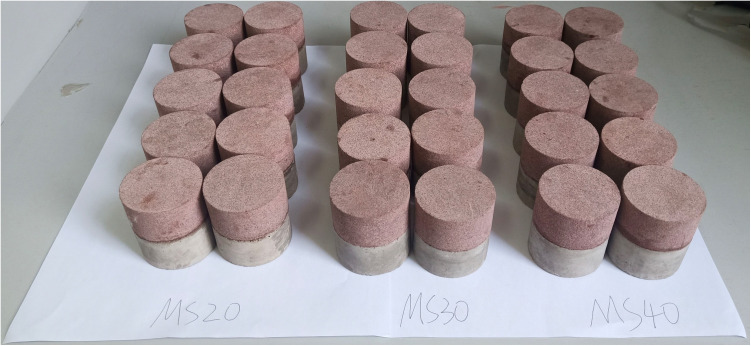
Testing specimens.

In order to make filling mortar-sandstone composite specimens, cement, sand and water were mixed together through different mix ratios. The preparation ratio of three materials are presented in Table 1. The mortar strength grade is set to three gradients, namely M20, M30 and M40. After the standard curing of mortar specimens was completed, both the mortar and sandstone specimens were machined into multiple cylindrical units and polished to process the samples into the required size. The size of mortar and sandstone specimens is the same, with a diameter of 48 mm and a height of 25 mm. Finally, epoxy resin was applied to the mating surfaces of two materials to ensure effective bonding between the mortar and sandstone specimens. Epoxy resin is made from a mixture consisting of resin A and resin B with a weight ratio of 2:1. The rock used in this paper was red sandstone that was taken from a quarry in Jining, Shandong Province. The compressive strengths of epoxy resin and sandstone are 64.43 MPa and 54.8 MPa, respectively. Some sandstone-mortar specimens are shown in Fig 1. In the static compression tests, the mortar specimen is located above the sandstone specimen, and the rigid loading plate directly contacts the mortar specimen.

**Table 1:** Preparation ratio of mortar/(kg·m^-3^).

Mortar Strength	Water	Sand	Cement
M20	270	1450	380
M30	310	1450	455
M40	340	1450	570

**Fig 2:**
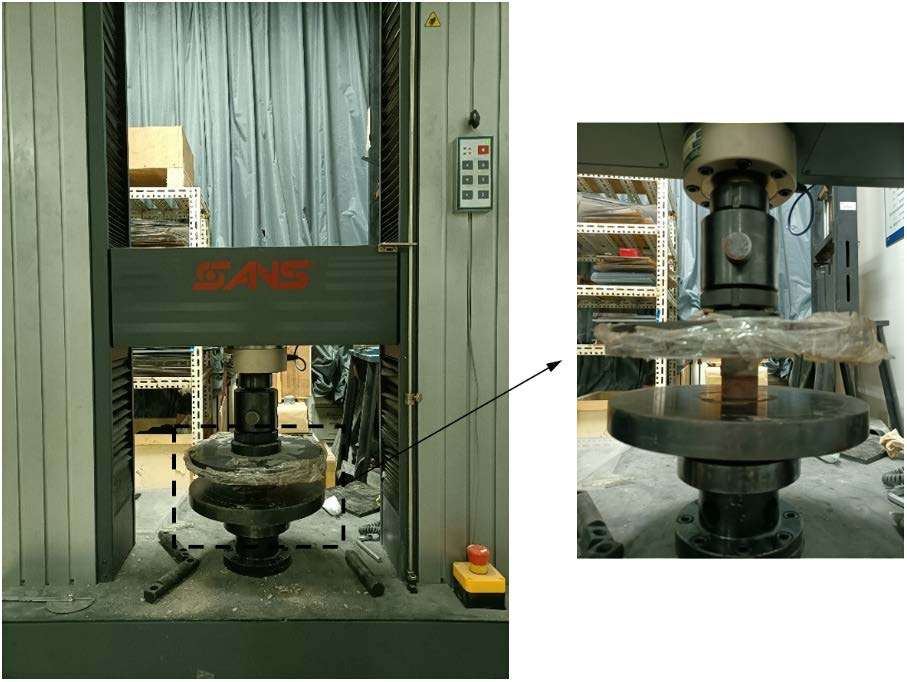
Servo pressure machine.

In this paper, the static uniaxial experiments adopt the electric uniaxial servo pressure testing machine, and the test device is shown in Fig 2. The test adopts displacement-controlled loading mode, and the uniaxial compression test is carried out at four loading rates of 0.02 mm/min, 0.06 mm/min, 0.2 mm/min and 0.6 mm/min for three different types of assemblies, respectively [21–22]. During the compression process, the uniaxial testing machine can automatically record the load-displacement curves of the specimens. By sorting out and analyzing the recorded load and displacement data, the stress-strain curves of the composite under four loading rates can be calculated.

## Results and analysis

### Stress-strain curves

**Fig 3:**
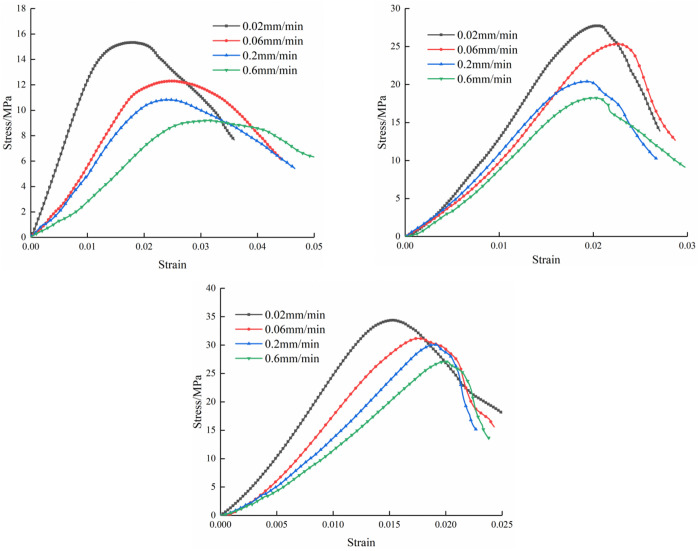
Stress-strain curves of composite.

Fig 3 shows the stress-strain curves of three specimens under different loading rates. As shown in Fig 3a, for the M20 specimen type, the stress-strain curves under four loading rates exhibit significant variations in amplitude. At the lowest loading rate of 0.02 mm/min, the curve demonstrates the steepest slope and fastest stress growth rate, whereas at the highest loading rate of 0.6 mm/min, the slope becomes minimal. Notably, the peak strength of M20 specimens shows a gradual decline with increasing loading rates. Fig 3b illustrates the behavior of M30 specimens, where the curves under four loading rates follow similar evolutionary trends. However, higher loading rates correlate with slower strength development. The stress-strain characteristics of M40 specimens in Fig 3c largely mirror those observed in M30 specimens. Overall, all three sandstone-mortar specimens display consistent trends in their uniaxial stress-strain characteristics under varying loading rates. These specimens exhibit ductile failure characteristics under uniaxial compression, primarily manifested by the gradual post-peak curve decline rather than abrupt brittle fracture. The M20 specimens, with lower strength, demonstrate pronounced rate-dependent characteristics, showing marked differences in stress-strain curve variations across loading rates. As specimen strength increases, the amplitude of stress-strain curve variations progressively diminishes, indicating reduced sensitivity to loading rate effects in higher-strength specimens.

### Strength parameter analysis

**Fig 4:**
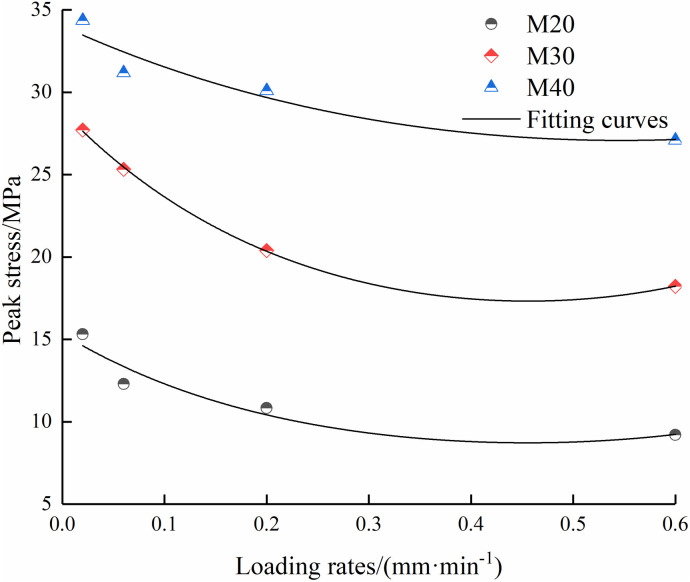
Relationships between peak stress.

**Fig 5:**
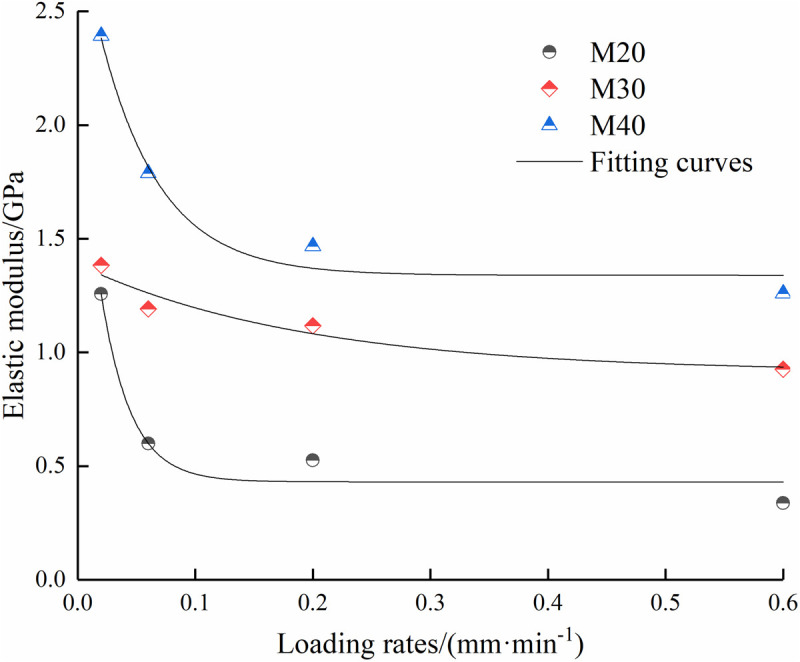
Relationships between elastic modulus and loading rates and loading rates.

As shown in Figs 4 and 5, the strength parameters of the composite specimens decrease with the increase of the loading rate. When the loading rate exceeds 0.2 mm/min, the peak strength enters into the slow decline stage, and the fitting curves tend to be gentle. Under four loading rates, the peak stress decreases by 39.9%, 34.2% and 21.2% when the mortar type is M20, M30 and M40, respectively, and the elastic modulus decreases by 53.1%, 33.2% and 47.3%, respectively. Because of the relatively small density of mortar, the three types of mortar can be categorized as soft rock due to their low strength class. The peak stress of soft rock gradually drops as the loading rate rises since the larger pores and cracks in the specimen have enough time to be compacted when the loading rate is low. Therefore, a larger stress is required to prompt the initial cracks to continue to expand. When the loading rate is high, the pores and cracks close rapidly in a short time. Therefore, the specimen is more likely to expand and connect into multiple penetrating crack surfaces, leading to a decrease in the peak stress. In addition, increasing the mortar strength has an obvious promotion effect on improving the peak stress.

### Energy evolution analysis

Under different loading rates, filling mortar-sandstone composite specimens show different mechanical responses, which are tightly connected to the energy development mechanism inside the specimen. The absorption and release of energy have an important impact on the destruction of the specimen. Therefore, in order to reveal the relationship between the energy development and static mechanical behavior of the composite specimen, it is imperative to examine the energy evolution process and different energy indexes. Under static compression, elastic energy and release energy are important components of specimen energy, and the calculation formulas of three kinds of energies are as follows [23–24].


$$U=\int_{0}^{\varepsilon_{u}}\sigma d\varepsilon$$ (1)

$$U_{e}=\frac{\sigma^{2}}{2E_{0}}$$ (2)

$$U_{d}=U-U_{e}$$ (3)

where *ε*_u_ is the limit strain, *E*_0_ is the initial elastic modulus, *U*, *U*_e_ and *U*_d_ are total strain energy, elastic strain energy and dissipated strain energy, respectively.

**Fig 6:**
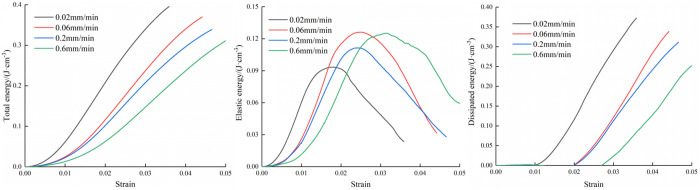
Variation curves of three types of energy of M20 specimen.

**Fig 7:**
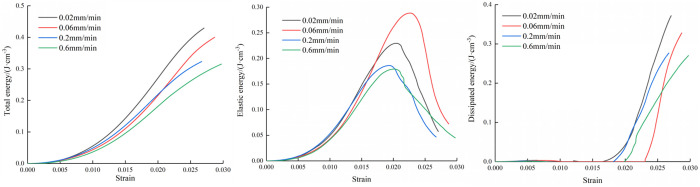
Variation curves of three types of energy of M30 specimen.

**Fig 8:**
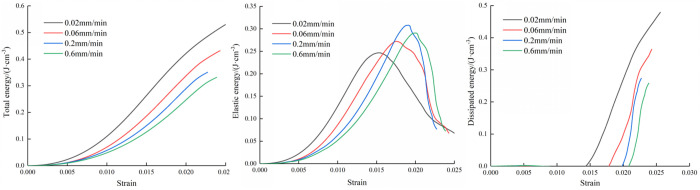
Variation curves of three types of energy of M40 specimen.

Figs 6–8 depict the energy development of the three types of composite at varying loading rates. The figures reveal that as strain increases, the total energy also increases. Additionally, the total energy displays a gradual increase during the early stages of loading, and the total energy difference is small at different loading rates. After loading to the elastic stage, the total energy increases rapidly until the specimen is destroyed. However, after reaching the peak stage, the total energy increase began to decrease. During the loading process, the combined specimen stores a portion of the total energy as elastic energy, while the remaining energy dissipates as surface plastic deformation and fracture development.

The trend of elastic energy variation mirrors that of the stress-strain curve. As the load intensifies, elastic energy steadily rises. Subsequently, after reaching peak stress, the elastic energy gradually diminishes to various extents. However, the elastic energy at the peak time is not different with the corresponding peak stress order. This is because in the calculation process of elastic energy, the elastic modulus is the denominator, and the size of the elastic modulus will directly affect the size of the elastic energy.

The dissipation energy is basically zero in the early period, and the variation range is very small. When the load is loaded near the peak stress, the dissipation energy increases explosively. This is due to the complete destruction of the specimen and the energy is released. When the mortar type is M20, the inclination of the dissipation energy growth curve is about 45° ~ 60°, however, the inclination of the dissipation energy growth curve is higher, about 70° ~ 80° in the case of mortar type of M30 and M40,

The evolution process of the three kinds of energy throughout the process is described in Figs 6–8. It can be seen that the three kinds of energy curves change obviously near the peak stress, which indicates that the peak point has an immeasurable impact in the energy evolution process. There is a big difference in the energy evolution law between the two stages before and after the peak. Therefore, it is necessary to further explore the energy development rules before and after the peak of the specimen to reveal the static mechanical response mechanism of the specimen.

In order to quantitatively describe the relationships between the mortar type and energy evolution, the values of total energy and energy before and after the peak were calculated. The figure shows the energy change curves of the three types of specimens at four loading rates.

Because the dissipated energy before peak is very small, the elastic energy occupies the main position, so the energy before peak characterizes the energy absorbed by the specimen from the outside in the pre-peak stage. Because the specimen can not absorb energy from the outside in the stage after peak, the specimen mainly releases energy. The more energy is released, the greater the destruction situation of the specimen is. Therefore, this paper uses post-peak energy to characterize the damage degree.

**Fig 9:**
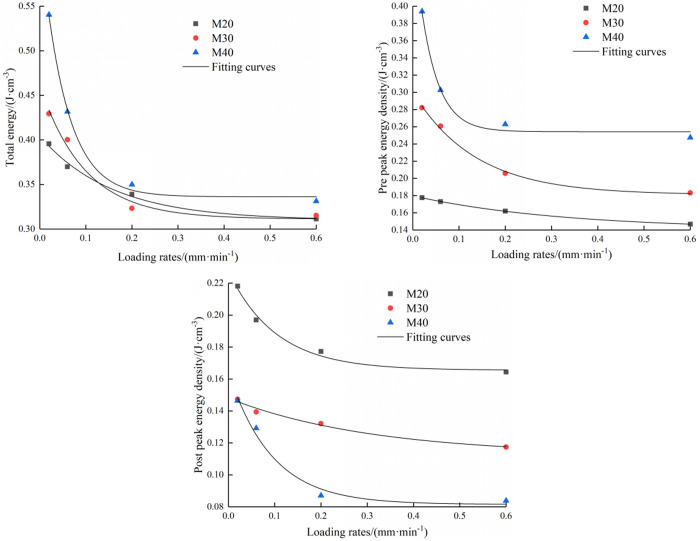
Relationships between total energy(a), pre-peak energy(b), post-peak energy(c) and loading rates.

Fig 9 shows that as the loading rate rises, the three types of energy decrease exponentially. In the case of loading rate of 0.002 mm/min ~ 0.2 mm/min, the three kinds of energy decrease greatly. When the loading rate exceeds 0.2 mm/min, the energy change range is small. At this time, the pores and cracks are quickly closed in a short time, which makes the specimen more likely to have a penetrating fracture surface, resulting in a gentle energy change curve. Under four loading rates, when the mortar type is M20, M30 and M40, the pre-peak energy decreases by 17.3%, 35.1% and 37.2% respectively, and the post-peak energy decreases by 24.6%, 20.3% and 42.7% respectively.

In the case of mortar type of M40, the total energy and pre-peak energy are higher than those of M30 and M20 specimens, and the post-peak energy is lower than the other two cases. With increasing in mortar strength grade, the ability to withstand loads of the specimen increases, and the specimen needs to absorb more energy during the mechanical process. Because the peak stress of the specimen with mortar strength of M40 is large and the high completeness of the specimen, the energy released in the post-peak stage is small.

### Energy dissipation damage model

#### Establishment of constitutive model.

The variation in damage evolution across loading rates is fundamentally governed by energy dissipation mechanisms, wherein crack propagation patterns dictate structural degradation through progressive loss of material integrity. The energy change will lead to the macroscopic and microscopic damage of the specimen, which will cause damage. In addition, the dissipated energy is closely linked to the damage because the elastic energy primarily resides within the specimen. Therefore, based on the dissipated energy of the specimen, a damage model suitable for the rock-mortar specimen is established.

It is well known that the relationship between stress and damage factor is [25]:


σ=Eε(1−D) (4)

where *D* is damage factor.

During the initial stage of loading, the specimen undergoes a compaction process, gradually becoming denser. Liu et al. [26] conducted static test research on composite specimens and introduced the definition of the compaction coefficient *K* when establishing the damage model. This coefficient accurately characterizes the mechanical behavio. Therefore, in this paper, the compaction coefficient is also used to analyze the damage characteristics of the specimen more comprehensively when establishing the damage model. The formula of compaction coefficient is as follows:


K=ln(aε+b) (5)

Where, *K* is compaction coefficient, *a* and *b* are fitting parameters.

Therefore, the relationship between damage factor and stress is:


σ=KEε(1−D) (6)

By applying the damage theory and statistical principles, the specimen under static loads can be viewed as comprising numerous small units. The damage factor of the specimen represents the ratio of broken small units to the total number of small units. In addition, this paper combines the dissipated energy under static load with the damage factor [27]. Therefore, in this paper, it is assumed that the statistical probability density relationship of the generalized Weibull distribution is satisfied between the number of small failure elements and the dissipative energy under static load. The formula is as follows:


$$P\left(U_{d}\right)=\frac{m}{k}\frac{U_{d}^{m-1}}{k}\times \exp\left(-\frac{U_{d}^{m}}{k}\right)$$ (7)

Where *P* is the probability density function of generalized Weibull distribution; *k* and *m* are the scale parameter and shape parameter, respectively.

With increasing in dissipation energy, the number of micro-units destroyed by the specimen itself under static load is:


$$n=\int_{0}^{U_{d}}NP\left(U_{d}\right)dU_{d}=N\left[1-\exp\left(-\frac{U_{d}^{m}}{k}\right)\right]$$ (8)

Where *N* and *n* are the total number of micro-units and the number of destroyed micro-units of the specimen, respectively.

The damage factor under static load can be expressed as:


$$D=\frac{n}{N}=1-\exp\left(-\frac{U_{d}^{m}}{k}\right)$$ (9)

By substituting Eq (5) and Eq (9) into Eq (6), the energy dissipation damage constitutive expression of the combined specimen can be obtained:


$$\sigma =E\varepsilon \ln\left(a\varepsilon +b\right)\exp\left(-\frac{U_{d}^{m}}{k}\right)$$ (10)

#### Model verification.

**Fig 10:**
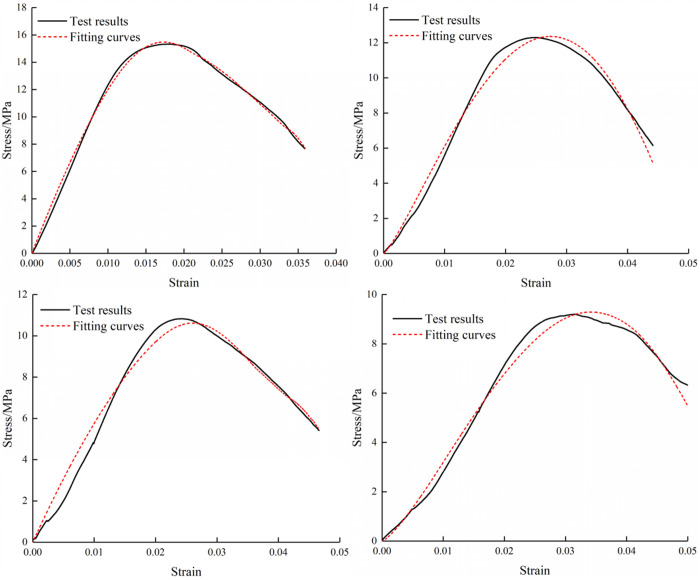
Fitting Stress-Strain Curves of M20 Specimen.

**Fig 11:**
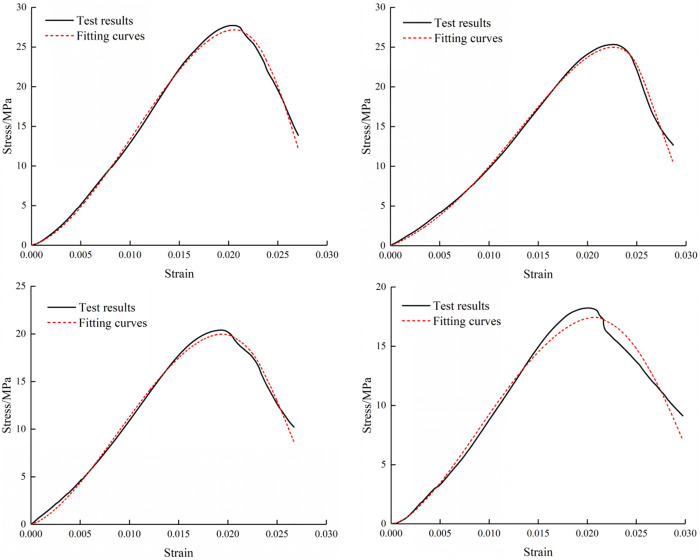
Fitting Stress-Strain Curves of M30 Specimens.

**Fig 12:**
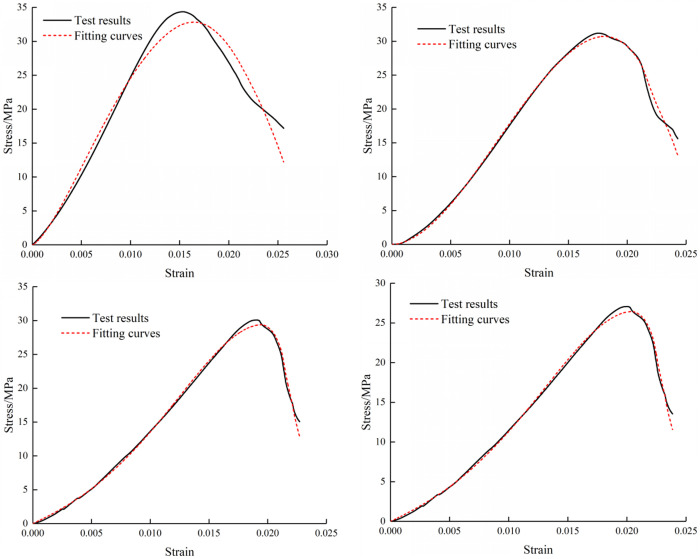
Fitting Stress-Strain Curves of M40 Specimens.

The stress-strain relationships of composite under four loading rates were obtained by nonlinear fitting. Because there are four parameters in the damage expression, fitting analysis was employed to solve these parameters in a nonlinear manner. The parameter values for all cases are presented in Table 2. As shown in Figs 10–12, the stress-strain curves and fitting curves at four conditions exhibit high similarity. Therefore, it can be considered that the damage model established is suitable for describing the failure characteristics of the composite subjected to diverse loads.

**Table 2:** Fitting parameters of damage model.

Specimens	*v*/mm·min^-1^	*a*	*b*	*m*	*k*	*R* ^2^
M20	0.02	−0.521	1.314	0.258	−0.085	99.7
	0.06	−0.423	1.021	0.032	−0.217	98.6
	0.2	−0.458	1.344	0.503	−0.003	98.8
	0.6	−0.663	1.039	0.057	−0.234	98.9
M30	0.02	−0.563	1.170	0.191	−0.273	99.7
	0.06	−0.148	1.469	0.361	−0.325	99.7
	0.2	−0.397	1.118	0.141	−0.257	99.6
	0.6	−0.114	1.004	0.059	−0.140	98.5
M40	0.02	−0.016	1.000	0.028	−0.116	97.5
	0.06	−0.251	1.000	0.087	−0.149	99.1
	0.2	−0.311	1.767	0.571	−0.250	99.8
	0.6	−0.294	1.763	0.569	−0.243	99.8

#### Failure modes.

**Fig 13:**
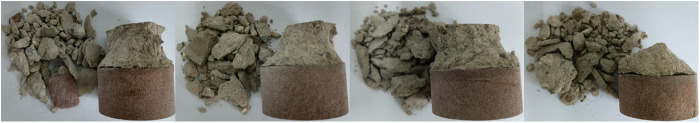
Failure Modes of M20 Specimens.

**Fig 14:**
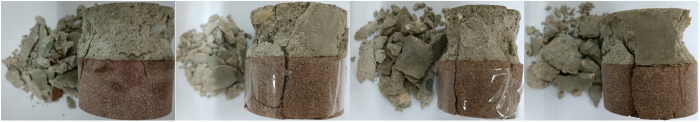
Failure Modes of M30 Specimens.

**Fig 15:**
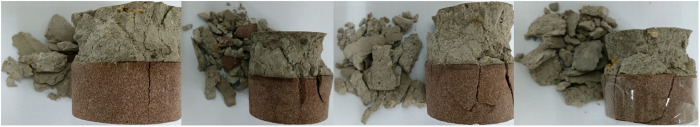
Failure Modes of M40 Specimens.

Figs 13–15 demonstrate the failure modes of the composite at four loading rates. In the case of mortar type of M20, the mortar specimen is crushed, part of which continues to bond with the sandstone through the epoxy resin, and the other part is crushed into fragments of different sizes, and the sandstone does not cause significant damage. In the case of mortar type of M30, the specimen has obvious splitting failure, and there is an obvious penetrating crack in the composite. The rock and mortar specimens were damaged to varying degrees, and spalling damage occurred in some areas of the mortar. Compared with the specimen of M20, the number of fragments after crushing of the mortar specimen decreased and the block size became larger. There is a macroscopic crack in sandstone. In the case of mortar type of M40, splitting failure is still the main form. The mortar specimens were damaged to a certain extent, and the number of fragments was further reduced. Compared with the specimens of M20 and M30, the integrity of the mortar specimens was higher. There are two macroscopic failure cracks in sandstone. It can be seen that the mortar strength has a undeniable impact on the failure characteristics of the composite. In addition, with increasing in loading rate, the energy of the rigid loading plate increases, the crushing degree of the mortar is greater, and the macroscopic crack propagation length in the sandstone is larger, resulting in a greater degree of damage to the specimen.

### Numerical simulation analysis

#### Numerical model and microscopic parameters.

**Fig 16:**
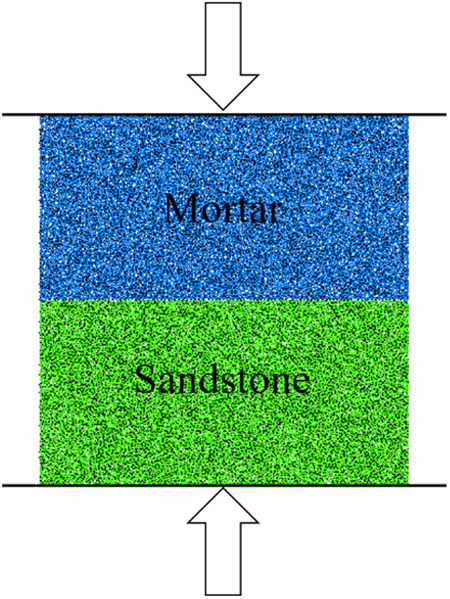
Numerical model for mortar-sandstone specimen.

The failure process of the composite specimen is gradual rather than instantaneous, involving continuous evolution of cracks. During the test, it is challenging to observe cracks at different times, and the stress distribution inside the specimen is also difficult to obtain. Consequently, in order to gain a more precise understanding of the failure process, the use of numerical analysis is a necessary choice. In this study, PFC^2D^ was utilized to set up a model of the combination, and the dimensions of the model and specimen are made consistent. The numerical model is depicted in Fig 16.

**Fig 17:**
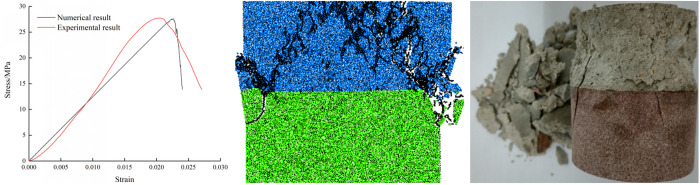
Comparison of experimental and numerical results.

In order to explore the crack evolution of specimens under different stress states, this paper selects specimens with mortar strength grade M30 to carry out static numerical analysis under different loading rates. Trial-and-error method is often used in PFC^2D^ simulation [28–31]. To calibrate the microscopic parameters, experimental data was used when the loading rate is 0.02 mm/min. By analyzing the data, the corresponding microscopic parameters were obtained. These calibration results are presented in Fig 17. It can be observed that the two results are essentially identical, indicating that the used parameters can be applied in the subsequent analysis. Table 3 presents the microscopic parameters.

**Table 3:** Micro-parameters of mortar and sandstone.

Micro-parameters	Values of Mortar	Values of Sandstone
Minimum radius of the particle (mm)	0.2	0.2
Ratio of maximum to minimum of radius	1.5	1.5
Young’s modulus of the particle (GPa)	0.42	0.46
Young’s modulus of the parallel bond (GPa)	0.42	0.46
Parallel-bond normal strength (MPa)	8	10.79
Parallel-bond shear strength (MPa)	8	12.22
Friction coefficient	0.6	0.6
Density of the particle (kg/m^3^)	2000	2500

#### Crack propagation process.

**Fig 18:**
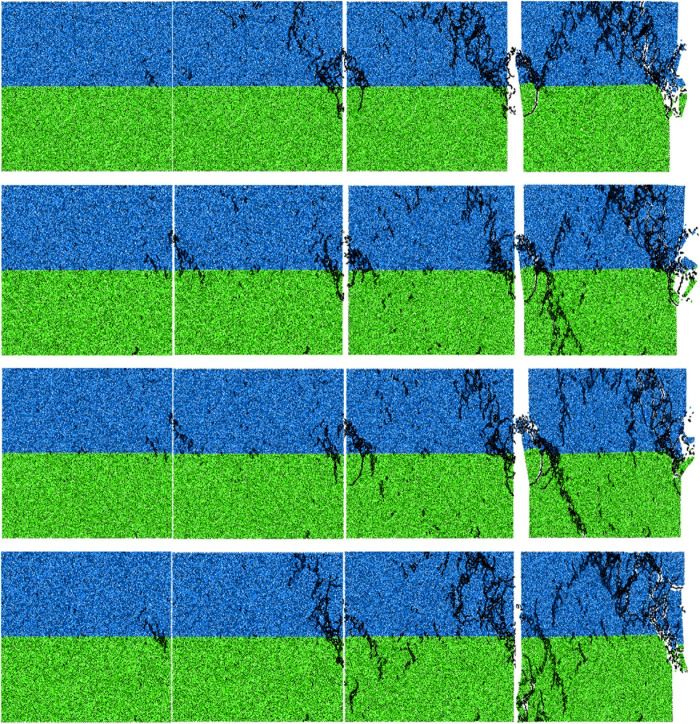
The crack propagation process of the M30 specimen under different loading rates.

From Fig 18, it is evident that the process of crack propagation can be categorized into four distinct stages: crack initiation stage, initial propagation stage, accelerated propagation stage, and failure stage. In the crack initiation stage, the positions where cracks start are essentially identical for all four loading rates, occurring at the two ends of rock and mortar. The crack first expands into the interior of the mortar. At this point, the specimen exhibits minimal damage, with only a small number of cracks primarily existed on the lower right side of the mortar. During the initial propagation stage, in the case of loading rate of 0.02 mm/min and 0.6 mm/min, the crack expands in approximately a 60° angle direction relative to the horizontal direction, eventually reaching the top of the specimen. In the other two cases, the crack does not extend too long after the crack initiation, and the inclined crack appears on the lower left side of the mortar. There are already scattered micro cracks in the mortar, but the number is small. The cracks under the four loading rates have one thing in common, that is, after the crack initiation, the crack propagates downward through the interface and forms an inclined crack in the upper right corner of the sandstone.

In the accelerated propagation stage, in the case of loading rate of 0.02 mm/min and 0.6 mm/min, the secondary cracks continue to propagate from the crack initiation position towards the top of the mortar, resulting in an increasing number of cracks and crushing of the lower left corner of the mortar. Compression cracks appear and expand to sandstone. In the other two cases, the crack started from the lower right corner of the mortar continues to expand to the top of the mortar. The crack on the lower left side of the mortar extends through the interface to the sandstone, forming a small broken zone in the middle of the composite. The number of cracks in the mortar increased further, and the micro cracks began to be scattered in the sandstone.

In the failure stage, the crack propagates extremely fast, and the crack at the lower left corner of the mortar eventually expands to the top of the specimen, which is approximately inverted V-shaped with the right crack. When the loading rate is 0.02 mm/min, the sandstone has a small number of cracks, and only a small crushing zone appears at the corners near the interface. In the other three cases, the crack in the corner of the mortar extends to the bottom of the sandstone, forming a macroscopic failure surface in sandstone. There are more internal cracks in the mortar than in the sandstone.

#### Stress field evolution.

**Fig 19:**
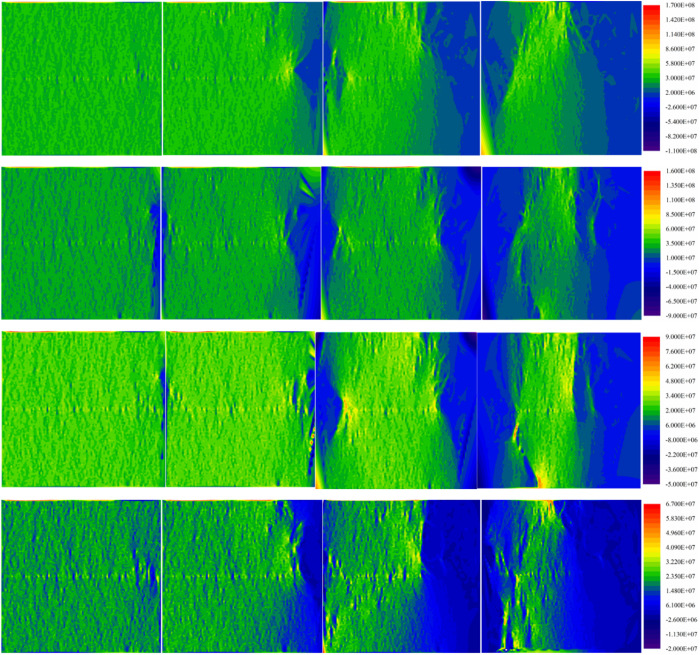
The stress evolution process of the M30 specimen under different loading rates.

As depicted in Fig 19, During the crack initiation stage, the stress in the specimen is predominantly compressive and exhibits a uniform distribution. However, the position where the crack initiates experiences a concentration of tensile stress, albeit at a small magnitude. In the initial expansion stage, the internal compressive stress of mortar and sandstone increases. The region of tensile stress primarily exists on the right side of the specimen. In the case of loading rate of 0.06 mm/min and 0.2 mm/min, a small part of the tensile stress area appears on the left side of the specimen. In the accelerated propagation stage, the compressive stress on the right side gradually decreases, resulting in a further increase in the tensile stress. Within the specimen, compressive stress concentration is observed at the corners near the interface, resulting in a small crushing zone. As the failure stage progresses, the specimen gradually loses its ability to bear load, causing a decrease in compressive stress on both sides. Nonetheless, a significant amount of compressive stress persists in the undamaged central area of the specimen.

The crack was first observed at the interface, indicating that the interface between different materials is a weak surface and is prone to damage. Furthermore, as the specimen is being loaded, it experiences pressure parallel to the axial direction. Because the diameter and height of the specimen are very close, both sides of the specimen are subjected to a certain tensile force, which leads to the crack initiation mainly distributed on both sides, and only casuing a small number of cracks in the middle. At the same time, it can be explained that the tensile stress in the specimen is the cause of crack propagation. The cracks continue to expand from both sides to the middle, the bearing capacity of the crack distribution area continues to decrease, resulting in a continuous decrease in the compressive stress. Since the cracks are mainly distributed on both sides, there is no large damage in the middle of the specimen, and it still has ability to bear loads. Therefore, there is a large compressive stress area in the specimen.

#### Crack initiation stress and crack number.

**Fig 20:**
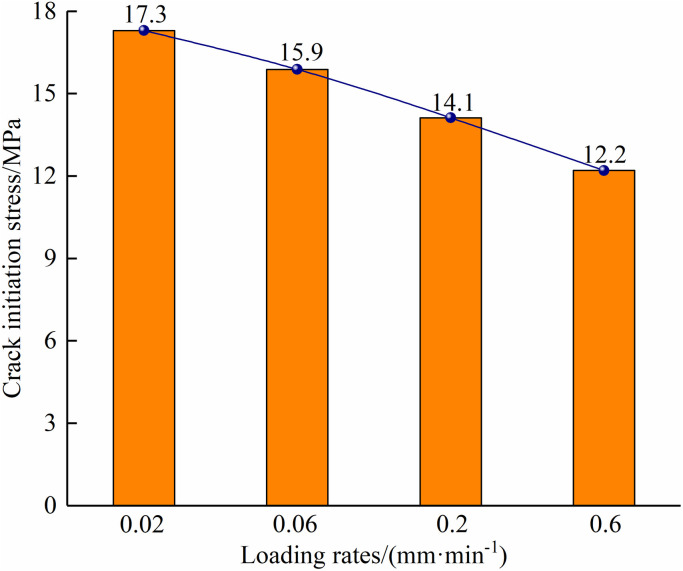
Relationships between crack initiation.

As depicted in Fig 20, the crack initiation stress drops as the loading rate rises. Specifically, the crack initiation stress decreases by 29.5% at four loading rates. Simultaneously, with increasing in loading rate, the pressure on the specimen also increases, which leads to the increase of the tensile force on both sides of the specimen. In addition, the interface between mortar and sandstone is a weak surface, which makes the crack more likely to appear, so the crack initiation stress gradually decreases.

**Fig 21:**
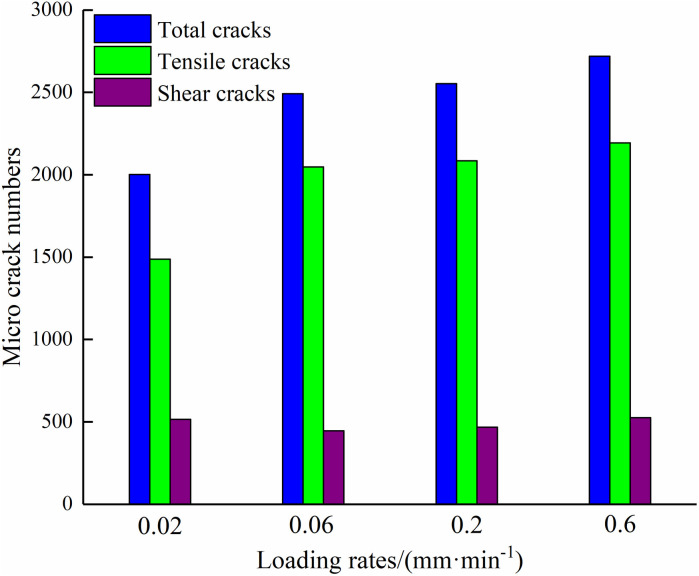
Relationships between microcrack stress and loading rates numbers and loading rates.

Fig 21 illustrates the change of the number of microcracks under four loading rates. The total number of cracks continuously rises as the loading rate increases, among which tensile cracks occupy the main position, and the number of shear cracks has little difference. At loading rates of 0.02 mm/min and 0.6 mm/min, the minimum and maximum numbers of cracks recorded are 2002 and 2719, respectively.

## Conclusions

In this study, uniaxial tests with four loading rates were conducted for the composite specimens to analyze the effects of loading rate on the mechanical parameters, energy evolution and fracture modes, and PFC2D was used to simulate and analyze the specimens with mortar grade of M30 to investigate the crack propagation and stress evolution process at four loading rates. The main conclusions are as follows:

(1) It is observed that the strength parameters of the composite decrease exponentially with an increase in the loading rate. Specifically, under four loading rates, the specimen with a mortar strength of M20 demonstrates the largest decrease in peak stress and elastic modulus, which amount to 39.9% and 53.1% respectively.
(2) With an increase in loading rate, there is an exponential decrease in three types of energy. More specifically, under four loading rates, the specimen with mortar type of M40 experiences the greatest reduction in pre-peak energy (37.2%) and post-peak energy (42.7%). Additionally, in the case of mortar type of M40, the total energy and pre-peak energy are higher, while the post-peak energy is lower compared to the other cases.
(3) Loading rate and mortar strength significantly affect the damage mode. The primary failure mode observed is splitting failure, and the damage degree of mortar is higher than that of sandstone. An increase in loading rate leads to greater crushing of the mortar and larger macroscopic crack propagation length in the sandstone. As for the mortar strength grade, an increase results in fewer fragments but a higher number of macroscopic damage cracks.
(4) Tensile stress is the main driving force for crack propagation. Initially, cracks are observed at the interface, and after crack initiation, cracks predominantly distribute on both sides of the specimen, with a smaller number of cracks in the middle region. In the early loading stage, stress distribution is relatively uniform, but in the later stages, there is a significant area of compressive stress in the middle of the specimen.
(5) Under four loading rates, the crack initiation stress decreases by 29.5%. Additionally, as the loading rate increases, the total number of cracks increases, and the tensile cracks occupy the main position. The minimum and maximum number of cracks observed are 2002 and 2719, respectively.

## Supporting information

S1 File Contains all data. (ZIP)
